# Systems Biology of the *qa* Gene Cluster in *Neurospora crassa*

**DOI:** 10.1371/journal.pone.0020671

**Published:** 2011-06-14

**Authors:** Xiaojia Tang, Wubei Dong, James Griffith, Roger Nilsen, Allison Matthes, Kevin B. Cheng, Jaxk Reeves, H.-Bernd Schuttler, Mary E. Case, Jonathan Arnold, David A. Logan

**Affiliations:** 1 Department of Physics and Astronomy, University of Georgia, Athens, Georgia, United States of America; 2 Genetics Department, University of Georgia, Athens, Georgia, United States of America; 3 College of Agricultural and Environmental Sciences, University of Georgia, Athens, Georgia, United States of America; 4 Statistics Department, University of Georgia, Athens, Georgia, United States of America; 5 Department of Biological Sciences, Clark Atlanta University, Atlanta, Georgia, United States of America; Technical University of Denmark, Denmark

## Abstract

An ensemble of genetic networks that describe how the model fungal system, *Neurospora crassa*, utilizes quinic acid (QA) as a sole carbon source has been identified previously. A genetic network for QA metabolism involves the genes, *qa-1F* and *qa-1S*, that encode a transcriptional activator and repressor, respectively and structural genes, *qa-2*, *qa-3*, *qa-4*, *qa-x*, and *qa-y*. By a series of 4 separate and independent, model-guided, microarray experiments a total of 50 genes are identified as QA-responsive and hypothesized to be under QA-1F control and/or the control of a second QA-responsive transcription factor (NCU03643) both in the fungal binuclear Zn(II)2Cys6 cluster family. QA-1F regulation is not sufficient to explain the quantitative variation in expression profiles of the 50 QA-responsive genes. QA-responsive genes include genes with products in 8 mutually connected metabolic pathways with 7 of them one step removed from the tricarboxylic (TCA) Cycle and with 7 of them one step removed from glycolysis: (1) starch and sucrose metabolism; (2) glycolysis/glucanogenesis; (3) TCA Cycle; (4) butanoate metabolism; (5) pyruvate metabolism; (6) aromatic amino acid and QA metabolism; (7) valine, leucine, and isoleucine degradation; and (8) transport of sugars and amino acids. Gene products both in aromatic amino acid and QA metabolism and transport show an immediate response to shift to QA, while genes with products in the remaining 7 metabolic modules generally show a delayed response to shift to QA. The additional QA-responsive cutinase transcription factor-1β (NCU03643) is found to have a delayed response to shift to QA. The series of microarray experiments are used to expand the previously identified genetic network describing the *qa* gene cluster to include all 50 QA-responsive genes including the second transcription factor (NCU03643). These studies illustrate new methodologies from systems biology to guide model-driven discoveries about a core metabolic network involving carbon and amino acid metabolism in *N. crassa*.

## Introduction

Systems biology provides a new paradigm to understand complex traits, such as carbon metabolism [1]–[6], homeostasis [7]–[8], development [7], response to environmental change [9], longevity [10], the clock [11], and life itself [12]. This new approach has a number of common elements [1], [3], including viewing living systems as biochemical and regulatory networks, measuring a system-wide response with genomic approaches as in RNA and protein profiling [14]–[15], and cycling through a rationalized discovery process to identify the true underlying network explaining a complex trait of interest [1], [3], [16]. One application of this approach is being exploited to identify how DNA sequence variation elucidates molecular networks that cause disease [17]. The challenge is that systems biology approaches are still in their infancy and require careful evaluation of their utility on particularly well studied systems.

Ideker *et al*. [1] chose one early paradigm for eukaryotic gene regulation, the *GAL* genes in *Saccharomyces cerevisiae* [18] to develop and test new approaches in systems biology. We have chosen another early paradigm for eukaryotic gene regulation, the *qa* gene cluster in *Neurospora crassa* [19] to develop and test these new systems biology approaches. Both the *GAL* genes and *qa* cluster have been biochemically and genetically studied for more than 40 years. A wealth of molecular biology experiments are then available to specify detailed biochemical and regulatory network models or *genetic networks*, for short, for galactose and quinic acid (QA) metabolism [1]–[2]. The *GAL* genes provided a testing ground for new genomic scale methodologies for measuring relative mRNA and protein abundances and an iterative process of genetic network identification [15], [1]. The *qa* gene cluster provided a testing ground for ensemble methods of network identification containing many parameters and limited data and an iterative model-guided discovery process in genomics experiments called *Computing Life* [2], [16]. Here we examine some of the strengths and limitations of these approaches on the *qa* gene cluster.

In prior work we have identified a working genetic network model to describe how the *qa* gene cluster functions in the cell to metabolize quinic acid (QA) as a sole carbon source [2]. We followed this work with exploration of how widespread the response to shift to QA is on the transcriptome with microaray experiments and used additional microarray data to refine the ensemble of genetic network models describing the *qa* cluster behavior [5]. As in a previous study of the *GAL* genes [1], we found the effects of the *qa* cluster are widespread, involving a QA response by more than 100 known genes in varied functional categories including carbohydrate metabolism, protein degradation and modification, ribosome biogenesis, and amino acid metabolism [5]. The challenge in understanding both galactose (GAL) and QA metabolism is that other processes could elicit a similar response to shift to these carbon sources. How do we distinguish the effects of these other secondary processes from the main effects of shift to galactose or quinic acid? For example, neither GAL nor QA are preferred carbon sources and could elicit a starvation response on the part of the cell [20], thereby unleashing a whole cascade of stress responses unrelated to the direct effect of the non-preferred carbon source. There is also a need to reconcile the 997 and 895 genes responding to GAL and QA with the average number of targets per transcription factor of ∼38 found in *S. cerevisiae* [21]–[22].

In this work our goals are several fold. First we wish to identify all QA-responsive genes. Second, we wish to distinguish a QA-response from other ancillary responses, such as to starvation. Three, we wish to begin the more or less complete description of the *qa* genetic network as is beginning to be achieved in *Escherichia coli* for carbon metabolism [3]. Finally, we wish to evaluate the performance of some of the new systems biology tools for rationalized discovery about genetic networks in the cell used here to discover the role of the *qa* gene cluster in metabolism [16]. In particular, we wish to extend the ensemble method to operate on a genomic scale transcriptional network. To address this problem we have developed a parallelized version of the ensemble method for network identification as described under Materials and Methods.

### Genetic network model for the qa gene cluster

The *qa* gene cluster consists of 7 genes on linkage group VII of *N. crassa* [23]. Four of the genes are structural genes (*qa-2*, *qa-3*, *qa-4*, *qa-y*); one has an unknown function (*qa-x*); and two are regulatory genes (*qa-1F* and *qa-1S*). The genes *qa-1F* and *qa-1S* encode a transcriptional activator and repressor, respectively, [24] that turn on and turn off the *qa* gene cluster. The gene *qa-1F* gene product QA-1F activates all genes in the *qa* cluster, allowing the use of QA as a sole carbon and energy source. The cluster is also known to be linked to a parallel biosynthetic pathway in aromatic amino acid metabolism as well [25].

**Figure 1:**
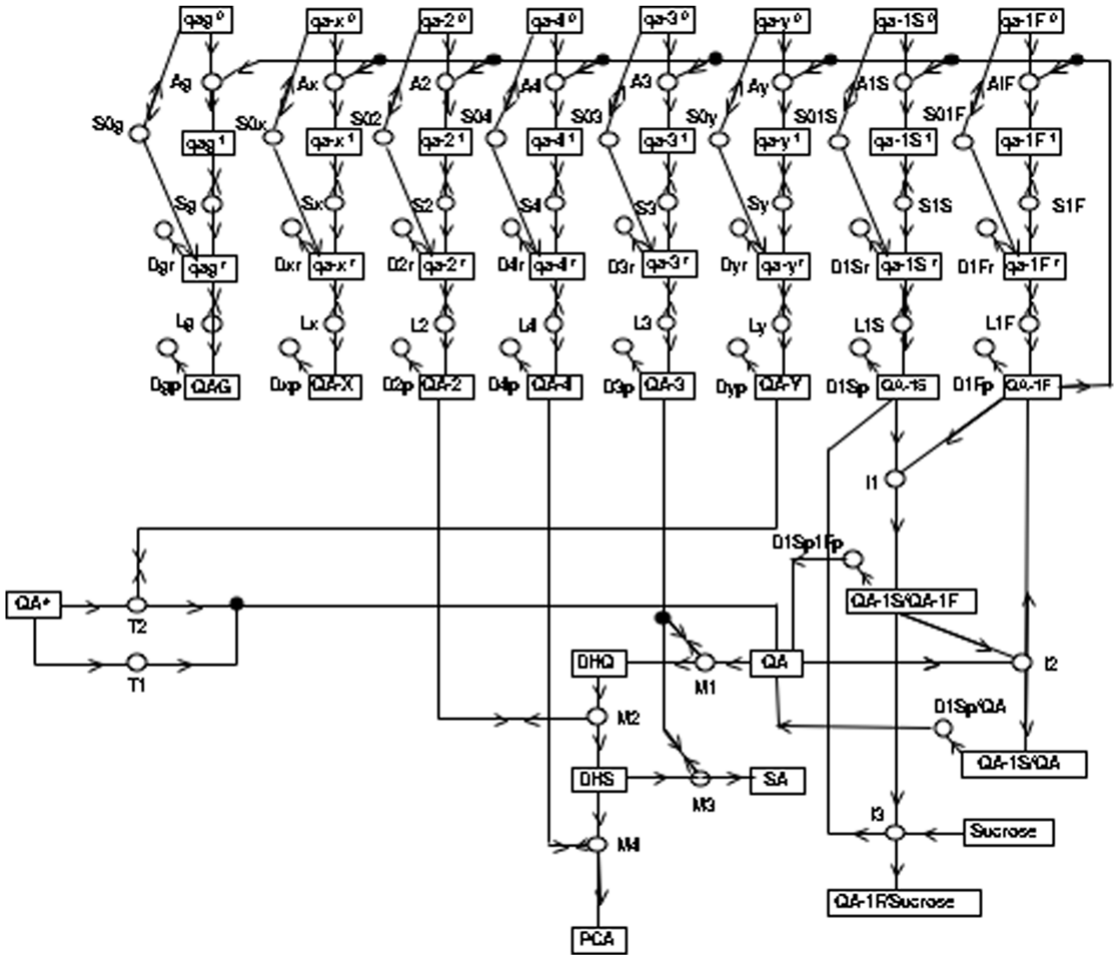
A genetic network for the *qa* gene cluster derived from [**2]**, [5****]. Molecular species (*i.e*., reactants or products) in the network are represented by boxes. The *qa-1F*, *qa-1S*, *qa-2*, *qa-3*, *qa-4*, *qa-x*, *and qa-y* gene symbols can be superscripted 0, 1, r0, r1, indicating, respectively, a transcriptionally inactive (0) or active (1) gene or a translationally inactive (r0) or active (r1) mRNA. Associated protein species are denoted with capital letters. Reactions in the network are represented by circles. Arrows entering circles identify reactants; arrows leaving circles identify products; and bi-directional arrows identify catalysts. The labels on each reaction, such as S_x_, also serve to denote the rate coefficients for each reaction. Reactions labeled with an S, L, or D denote transcription, translation, or degradation reactions, respectively. Reactions without products, such as D_xr_, are decay reactions. The rate constants specify the right hand side of the kinetics model. The figure differs from the network in Logan *et al*. [5] in that the 50 QA-responsive genes (*qag*) in Table 3 have been included under the control of QA-1F (model all1F-2E in Table 5).

This information enabled formulation of an initial detailed genetic network shown in **Figure 1** (minus the QA-responsive genes (*qag*) on the left hand side) that explains how QA metabolism functions [2], [5]. Following the notation of [26], circles denote reactions, and boxes, molecular species (*i.e*., genes, mRNAs, proteins, and metabolites) appearing in this chemical reaction network. Arrows entering a circle denote reactants, and arrows leaving a circle denote products. Double arrows indicate that a molecular species appears on both the left and right side of the reaction and is a catalyst. As an example, enzymes enter reactions with double arrows. The overall structure of this genetic network for carbon metabolism has the Central Dogma at the top and the biochemical pathway for QA metabolism, at the bottom. On the left side of the network is the transport process for QA involving the permease, QA-Y, and on the right side is the genetic regulatory mechanism involving the regulators, QA-1F as QA-1S, as well as metabolites hypothesized to affect these regulators.

In this network model, the QA-1F protein activates all of the *qa* cluster genes, including a gene (*qa-1S*) leading to its own inactivation, by means of the QA-1S repressor protein [27], [24]. The active *qa-1F^1^* gene is transcribed into its cognate mRNA *qa-1F^r^*, which in turn is translated into its cognate protein QA-1F. The QA-1F protein, in turn, activates all of the *qa* cluster genes in the A-reactions in **Figure 1** in a positive feedback loop. One of these is the *qa-1S* gene, encoding a repressor. The encoded QA-1S protein thereby counteracts QA-1F in the I1 reaction and shuts down QA-1F as QA-1S accumulates by sequestering QA-1F. The action of the QA-1S protein is facilitated in reaction I3 by a preferred carbon source, such as sucrose, binding to QA-1F, thereby providing a mechanism for catabolite repression. In addition, QA-1F may also activate a number of QA-responsive *genes (qag)* that serve as yet to be identified outputs of this circuit. The number of these *QA-responsive genes* in the genome, and hence the extent of QA control over metabolism is largely unknown [5].

**Figure 1** then specifies fully the null hypothesis for this paper with 204 rate constants and 147 initial molecular species concentrations as parameters. A number of alternative hypotheses to **Figure 1** will be considered. For example, one alternative hypothesis is catabolite repression by inducer exclusion [28].

### Predictions about QA-responsive genes from the genetic network

Six predictions about the behavior of QA-responsive genes can be made from the genetic network in **Figure 1** and prior work.

1. When WT cells are shifted from sucrose to quinic acid as described in Materials and Methods, the mRNA levels are predicted to respond [2]. This experiment is referred to as experiment **1-QA response** to identify a QA-response in WT. In contrast as a control, we can also predict that if WT cells were shifted from sucrose to sucrose, under the genetic network model there should be no response in mRNA levels by QA-responsive genes [2]. We will refer to this as the **control experiment**.
2. Since QA is a poor non-preferred carbon source, it is possible that cells will not differentiate a shift to QA from a shift to no carbon source, *i.e*., a starvation response. So, in experiment **2-starvation** we shift the cells from sucrose to Fries Medium without a carbon source [29] to differentiate a response to QA in experiment 1 from a response to starvation in experiment 2. The prediction is that genes under *qa* cluster control should not have a starvation response because there is no such mechanism in **Figure 1**.
3. The first two perturbation experiments represent environmental perturbations. The last **experiment 3-QA response by** ***qa-1F*** is a genetic perturbation. If *qa-1F* mutant cells were shifted from sucrose to QA, the genetic network would predict that there should be no QA-response [2] because some *qa-1F* mutants, such as the one selected in Materials and Methods, do not make functional transcriptional activator protein QA-1F. It is still possible that *qa-1F* mutant cells might perceive that the shift to a poor carbon source, such as QA, as a starvation signal, eliciting other “starvation” genes to respond in a *qa-1F* mutant. Alternatively, if there were alternative pathways for the metabolism of QA [30] or if there were other transcription factors that can substitute for QA-1F in function, we may see a response by genes in the absence of a functional *qa* gene cluster. The QA-1F protein is a member of the largest family of transcription factors in *N. crassa*, and such transcription factors are well known to act redundantly [31].
4. Another prediction that can be made about the dynamics of the mRNA levels of QA-responsive genes from the genetic network in **Figure 1** [2]. If we were to compare such a gene's mRNA level at time 0 in the shift from sucrose to QA to the average mRNA level at later times, we should see a dramatic rise in mRNA abundance with time. We search for this kind of change. This does not preclude the *qa* gene cluster acting to decrease expression of other genes, but if such a decrease were observed, it would have to be an indirect effect in the genetic network. For example, the QA-1F protein would need to target a repressor, for example, which in turn would down-regulate other genes under its control. This last prediction places an additional constraint on what are considered genes directly regulated by the *qa* cluster.
  These four microarray experiments and the expected dynamics of mRNA levels of genes under QA-1F control provide a means to sift through the 895 genes that respond to the initial shift experiment under experiment 1 [5]. A *QA-responsive gene* will be defined as one whose mRNA level: (1) in WT responds to shift from sucrose to QA; (2) in WT does not respond to shift from sucrose to Fries (*i.e*., no starvation response); (3) in a *qa-1F* mutant does not respond to shift from sucrose to QA; (4) in WT does not respond to shift from sucrose to sucrose (*i.e*., in the control experiment); (5) increases significantly from time 0 to later time points in WT when shifted from sucrose to QA.
5. There are two more predictions from prior work about QA-responsive genes. Case and colleagues [30] presented both genetic and biochemical evidence for the interrelationships of the QA and **shikimate** (***aro***) pathways. A mutation in *aro-1* (which converts dehydroshikimic acid (DHS)→shikimic acid (SA)) was suppressed by *qa-3^+^* in an *aro-1/qa-4* double mutant background. The double mutant leads to the accumulation of DHS, which by mass action allows the QA-3 protein to convert DHS to SA. The *aro-1* mutation was thus sidestepped. Also a block in the *aro* pathway via an *aro-9* mutation was demonstrated to lead to the internal induction of the *qa* cluster. The *qa* and *aro* pathways are coupled by redundancy of function and mass action. We thus predict that aromatic amino acid metabolism will have encoding genes that are QA-responsive.
6. It has been long known that sorbose-resistant mutants constitutively activated QA-metabolism on sucrose (M. E. CASE, unpublished results). We then expect that some of the QA-responsive genes will be sorbose-resistance mutants (*sor-1-sor-4* in [32] as well. We thus expect that starch metabolism should be represented among QA-responsive genes. (While fungi generally use glycogen instead of starch, we continue to refer to the “sucrose and starch metabolism” because that is the label of this metabolic module used in the Kyoto Encyclopedia of Genes and Genomes.

## Materials and Methods

### Strains

Strain 74A-OR23-1A is used in all shift experiments except experiment 3 (QA-response by *qa-1F* mutation). In experiment 3 a *qa-1F*, *pan-2* mutant named 125-23-1A (MEC, unpublished) is used to inactivate the entire *qa* gene cluster. Strain 125-23-1A was obtained from a cross of *pan-2,a* named B23 [33] with *qa-1F,A* named 125 [34]. Strain 125 was UV-induced in a 74A-OR23-1A background [34], and strain B23 was X-ray induced in the same background [33]. There is no known interaction between *pan-2* and the *qa* cluster (MEC, unpublished).

### Liquid growth conditions for harvesting RNAs

Establishment of liquid cultures follows [35]–[36]. A culture in a 250 ml flask with 50 ml of Fries medium [29] + sucrose (1.5%) + agar (1.5%) is inoculated, grown for 2 days at 30°C and then is shifted to room temperature under light for 5–6 days to induce conidiation. Conidia are suspended by adding 50 ml of Fries + sucrose (1.5%) to a flask. The suspension is added to a new 500 ml flask with 100 ml of Fries + Sucrose (1.5%). The new liquid cultures are grown at 25°C at 300 rpm overnight (14–16 hrs) on a shaker (New Brunswick Scientific, Edison, NJ, Series 25). Conidia are harvested through a Buchner filter funnel containing 541 Whatman filter paper, rinsed with distilled water and grown for 0, ½, 1, 1.5, 2, 4, 6, and 8 hrs in a new 500 ml flask with Fries + QA (0.3%). Conidia are harvested again as just described, squeezed of liquid, and frozen at −70°C for RNA harvesting. Fries medium is supplemented as needed for various *qa* cluster mutants used, such as *pan-2,qa-1F*.

### Experiment 1 (QA-response)

To identify QA-responsive genes (experiment 1) all liquid cultures are processed as described in the previous section with a shift from sucrose (1.5%) to QA (0.3%) for varying amounts of time on QA ranging from 0 to 8 hrs. Cells are placed at 70°C to await RNA harvesting using a High Pure RNA kit (Roche, Inc.). The harvested RNA is subjected microarray analysis as described below.

### Experiment 2 (Starvation response)

To study a starvation-response (experiment 2) the only change from experiment 1 is to shift to Fries medium with no carbon source (instead of Fries + QA (0.3%)) for 0, 0.5, 1, 1.5, 2, 4, 6, or 8 hrs.

### Experiment 3 (QA-response by qa-1F mutant)

To study the QA-response in a *qa-1F* mutant (experiment 3), the experiments are performed as in experiment 1 but with the *qa-1F*, *pan-2* mutant. Pantothenate Calcium (0.0002%) was used to supplement Fries medium.

### Control experiment

As a control, a shift experiment as in experiment 1-QA response is performed but with a shift from sucrose (1.5%) to sucrose (1.5%).

### RNA isolation

RNAs are isolated as described in [16].

### Design of 12K oligonucleotide arrays (Combimatrix, Inc.)

The arrays were constructed exactly as described in [16] with two changes. The same design was used on all Combimatrix chips here, and each chip had eight rDNA-derived oligonucleotides.

### RNA amplification and oligonucleotide array hybridization

750 ng of total RNA (as determined by a Nano LabChip (Agilent Technologies, Inc.)) is amplified and hybridized to oligonucleotide arrays (Combimatrix, Inc.) as previously described [16] with one change. Laser confocal scanning is performed on a GSI Lumonics ScanArray 5000 (now manufactured by Perkin-Elmer, Inc.) using two laser power and a photomultiplier (PMT) gain settings adjusted less than 10% between arrays from one of the two settings.

### Quality control on RNAs

RNA samples are verified to have a ratio of at least 1.00 and their profiles examined for 28S/18S rRNA on the LabChips (Agilent Technologies, Inc.) with a ratio of 2.00 being considered the best [37]. As shown in **Figure S1**, the quality of the RNAs is quite high with little sign of degradation, although the total amount of RNA used (750 ng) in amplification needed to be kept constant from sample to sample. Arrays hybridized to aRNAs are also visually scanned for trends in the foreground median count (described below) in control sequences in the (x,y) coordinates on arrays. In addition, using 4 λ-oligonucleotides spiked into each aRNA (see Methods in [16], the coefficient of variation (CV) in median foreground count for these spiked oligonucleotides is estimated, and if the chip has a CV greater than 0.65 (n = 88 because each quality control oligonucleotide is represented 22 times per microarray), the sample is usually not used and redone. Each oligonucleotide array is verified to have 50–52% of its features above median background (as determined from the 633 negative controls). This percentage (50–52%) of identified genes with expression above background is the same (51%) as for clock oligonucleotide array experiments on *N. crassa* [16] and in the range (38%–60%) of previously published oligonucleotide array experiments [38]–[39]. All samples passed these quality control steps. Data are deposited in the MIAME-compliant *Neurospora crassa* functional genomics database at http://www.yale.edu/townsend/Links/ffdatabase/introduction.htm [40] under accession numbers 134 (experiment 1-QA response), 133 (experiment 2-starvation), 135 (experiment 3-QA response by *qa-1F*), and 136 (control).

### Background subtraction and normalization

There are 3 perturbation experiments and one control experiment, each with 8 time points. Typically each experiment uses a different scanner. Each time point is associated with a single microarray which contains 12,544 features (12K chip) including 11,911 genes of interest and 633 negative control genes. All of the background subtraction and normalization is done within each of the 32 ( = 4 experiments×8 time points) microarrays. The background subtraction is accomplished by subtracting the minimum reading of all 11,911 genes of interest for each chip. All the data are then transformed by means of taking the base 2 logarithm and normalized in the following robust way:
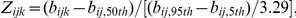
where *i* = 1,…,4; *j* = 1,…,8; *k* = 1,…,11911; *b_ijk_* is the background subtracted and base 2 logarithm transformed microarray value of the *k^th^* gene in the *j^th^* chip of the *i^th^* experiment; *b_ij,50th_*, *b_ij,95th_ b_ij,5th_* are the 50^th^, 95^th^ and 5^th^ percentile of the *b* values respectively within the *j^th^* chip of the *i^th^* experiment.

The reason that the sample median (*b_ij,50th_*) rather than the sample mean is used as a center is to guard against the effects of a few very large outliers, such as the QA-responsive genes. The denominator expression (*b_ij,95th_* - *b_ij,5th_*)/3.29 is a robust estimate of the standard deviation based on the middle 90% of the data points. If the data were truly normally distributed, this estimator would be approximately the sample SD of the *b* values. The reason the actual sample SD is not used is, as above, to guard against outlier effects.

### Least Median Squares (LMS) regression

After preliminary analysis of the positive controls (known genes from the *qa* gene cluster), we noticed that many of these positive controls (see results) had a very sharp increase from the first measured time point (*t* = 0) as compared to all other time points. Hence, we decided to try to identify other genes with this same sort of behavior. Consequently, to conduct this test for the four experiments, a scatter plot was made with the normalized *Z* values for all genes, where the x-axis displayed the *Z* value (*Z_i,1,k_*) at the first time point (*t* = 0) and the y-axis displayed the average value (*Z_i,avg,k_*) at the other seven time points, *i.e*.,
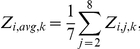
where *i* = 1, 2, 3, 4; *k* = 1, 2,…, 11911.

The four scatter plots are shown in the Results. In each case, as one can clearly observe, there is a positive linear association between the x and y variables. However, there are a few data points which deviate significantly from the linear pattern. Since these few outliers could distort a traditional ordinary least squares (OLS) regression fit, we also fit the least median squares (LMS) regression [41] line to each of the 4 experimental data sets. It happened that the OLS method was not as effective as LMS, thus resulting in a low power using the identification method described below. On the other hand the LMS method selected too many genes in Experiment 2 using the same method (see results). So finally we combined these two methods and took the average of the fitted LMS line and OLS line as our best predictor line. From this line we calculated the standardized residuals
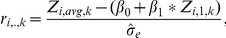
where *r_i,.,k_* is the standardized residual, *β*_0_ and *β*_1_ are the regression coefficients from the averaged OLS/LMS method, and 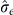 is the 75^th^ percentile of the absolute residual divided by 1.15; and calculated the p-values in the standard way 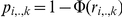, where Φ is the CDF of the standard normal distribution; These p-values yield the probability of a residual of the size observed or larger, given that the fitted line is correct. Of course, if we naively choose those points with p-values less than 0.10 as being significant, we will have too many “false positives” because of multiple testing. Hence, we used the Benjamini-Yekutieli False-Discovery Rate Correction procedure [42] with *α* = 0.10 to detect those genes which were truly significant. See the Results. As can be seen from Table 1, the false discovery rate procedure seriously restricted the number of genes that are truly considered to be on. In Table 2 we see the effect on choice of regression line.

**Table 1:** Number of genes identified with or without a Multiple Test Correction using a significance level of α = 0.10 [42].

	Naïve: Number of genes identified as ON	Multiple Correction Procedure: Number of genes identified as ON
Experiment 1	706	46
Experiment 2	1303	54
Experiment 3	376	2
Control Experiment	637	5
Identified QA Responsive gene	466	41

**Table 2 pone-0020671-t002:** Number of genes identified with ordinary least squares (OLS), with robust least median squares (LMS) [41] and with the averaged OLS/LMS method (see description in Materials and Methods) using a significance level of α = 0.10.

	OLS	LMS	OLS/LMS
Experiment 1	31	67	46
Experiment 2	12	253	54
Experiment 3	2	2	2
Control Experiment	3	13	5
Identified QA Responsive gene	28	50	41

### Microarray analysis

**Figure 2:**
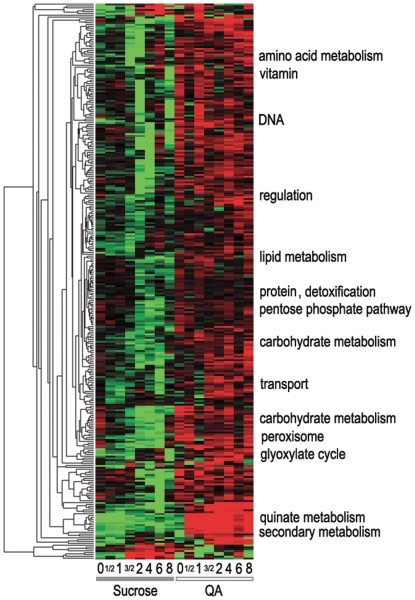
Transcriptional profile of 300 putative genes with QAREs upstream at 0, 1/2, 1, 3/2, 2, 4, 6, and 8 hrs after shift from sucrose (1.5%) to QA (0.3%) after background subtraction, normalization within arrays relative to the grand median of each chip, logging, and clustering with average linkage using Euclidean distance between mRNA profiles of different genes [44]. A total of 8 time points on sucrose (1.5%) are concatenated prior to the shift from the control experiment (see Materials and Methods). The bright green is −3, and the bright red is +3 is expression level on a decadic log scale. Data arose from 16 oligonucleotide arrays probed with a biotin labeled aRNA. Varied FUNCAT classifications are overlayed in the right margin of this microarray experiment [43]). The *qa* cluster genes in Fig. 1 are represented at least 5 times on each chip. The 300 genes with QAREs upstream were selected by a paired t-test applied to the profile of each of the 11,000 genes, and those with a significant t with t_7_>2.365 (α = 0.05) are displayed.

Each oligonucleotide array is typically scanned ∼50–60 times, and from these scans a median foreground (FG) count on each microarray feature is obtained for each of 12,544 (12K) features on an oligonucleotide array. From each median foreground count on an oligonucleotide array, a background subtraction is performed as described in the previous section. Then the median foreground counts are normalized within arrays as described in the previous section. A MIPS functional classification (FUNCAT in Table 3) is assigned to each feature on a chip [43]. Hierarchical clustering of genes is implemented in Cluster 3.0 [44] available at http://bonsai.ims.u-tokyo.ac.jp/~mdehoon/software/cluster. Options selected for analysis are average linkage (*i.e*., UPGMA) using phenotypic correlation on the Z_ijk_ because of its superior aggregate performance across a variety of cluster validation criteria [45]. Heat plots of trees as in **Figure 2** are constructed with Java TreeView 1.0.12 [46] available at http://treeview.sourceforge.net.

**Table 3 pone-0020671-t003:** QA-responsive genes are described by their NCU number [47], *N. crassa* gene name when available, MIPS FUNCAT classification [43]), KEGG assignment, whether or not they possess a known QA response element (QARE), and whether or not they are leaky in their response to quinic acid (QA).

NCU#	Feature ID	*Gene name*	FUNCAT	FUNCAT Description	KEGG	QARE	Leakiness$
1517	10032	*gla-1*	1.05.01.01.01	Sugar, glucoside, polyol, & carboxylate metabolism			13669
1830#	12383		1.01.09.05.02	Tyrosine degradation			3188
3643*	9441		11.02.03.04	Transcriptional control			4869
5499#	11438		1.01.09.05.02	Tyrosine degradation		X	1320
6024#	9636	*qa-4*	1.20.15	Biosynthesis of derivatives of dehydroquinic acid,…		X	**286**
2704*	12467		1.05.01	C-compound & carbohydrate utilization	Valine Metabolism		1327
6023#	8395	*qa-2*	1.20.15	Biosynthesis of derivatives of dehydroquinic acid,…		X	1533
6023#	11955	*qa-2*	1.20.15	Biosynthesis of derivatives of dehydroquinic acid,…		X	1971
8315#	10618		1.20	Secondary metabolism			1106
6675*	8082		1.05.01.01.01	Sugar, glucoside, Polyol, & carboxylate metabolism		X	205
5134#	8639		99	unclassified			7512
9491	8958	*fea-1*	1.05.01.01.02	Polysaccharide degradation		X	**270**
5627	8797	*ght-1*	20.01.03	C-compound & carbohydrate transport			4020
5897#	9998		20.01.03	C-compound & carbohydrate transport			3452
9525#	5569		99	unclassified			1446
2364#	12455		99	Unclassified (putative C2H2 domain)		X	15321
6025#	12398	*qa-3*	1.20.15	Biosynthesis of derivatives of dehydroquinic acid,…		X	1965
6881	1839		1.05.01	C-compound & carbohydrate utilization			22247
0801	5142		20.01.03	C-compound & carbohydrate transport			3540
6023#	6039	*qa-2*	1.20.15	Biosynthesis of derivatives of dehydroquinic acid,…		X	1356
4072#	7586		99	unclassified	Tryptophan metabolism	X	4949
6026#	7429	*qa-y ,distinct primer*	1.20.15	Biosynthesis of derivatives of dehydroquinic acid,…		X	2785
0591*	4627		01.01.11.04.02	Leucine degradation	Valine metabolism	X	4490
0992	7199		99	unclassified			9063
9133#	12401	*acw-7*	99	unclassified			70579
5755#	10391		99	unclassified			3023
6025#	5215	*qa-3*	1.20.15	Biosynthesis of derivatives of dehydroquinic acid,…		X	2897
6023#	9092	*qa-2*	1.20.15	Biosynthesis of derivatives of dehydroquinic acid,…		X	1448
6025#	5518	*qa-3*	1.20.15	Biosynthesis of derivatives of dehydroquinic acid,…		X	1718
8315#	3959		1.20	Secondary metabolism			1106
7888	9891		99	unclassified		X	8188
4914	9342		99	unclassified			2925
6023#	4289	*qa-2*	1.20.15	Biosynthesis of derivatives of dehydroquinic acid,…		X	1209
6524#	2391		16.09	Lipid binding			2529
6025#	4856	*qa-3*	1.20.15	Biosynthesis of derivatives of dehydroquinic acid,…		X	1221
6025#	3426	*qa-3*	1.20.15	Biosynthesis of derivatives of dehydroquinic acid,…		X	1875
5291	9604		14.07.04	Modification by acetylation, deacetylation		X	3365
6524#	12359 ,duplicate		16.09	Lipid binding			2529
0121#	8145		20.03.01.01	Ion channels			11429
5775	10122	*aap-13*	99	unclassified	Amino acid transporter		16077
6815	8623		40.01.03			X	8117
4872#	9829		99	unclassified			22898
5315#	12523		99	unclassified			17836
8541#	8022		99	unclassified			7732
1231	10269		20.03.01.01	Ion channels			**639**
8771	8084		1.01.09.04.01	Phenylalanine degradation			6392
10021* #	5062	*hgt-1*	20.01.03.01	Sugar transport	Monosaccharide transporter		**258**
3415	11884	*cbs-3*	32.05.01	Defense-related proteins		X	2498
9429	7934		99	unclassified	Flavin mono-oxygenase	X	1502
9873#	4944	*acu-6*	2.01	Glycolysis/glucanogenosis	Carbon metabolism	X	2970

The listed entries survived a test for being significantly (α = 0.10) above the LMS regression line in experiment 1 after a multiple-test correction (see Materials and Methods and Table 2). Entries significant at the α = 0.05 have their NCU number footnoted with a #. The *gla-1* gene is probably a sorbose-resistant mutant in the classic sense [32] and *acu-6* encodes a phosphenolpyruvate carboxykinase [32]. The high-affinity glucose transporter (*hgt-1*) was first characterized by XIE *et al*. [20]. While another gene designated by NCU03415 is classified as defense-related, it would also appear to encode a function in fermentation and glycolysis as an aldehyde dehydrogenase. Genes with NCU06881 and NCU02704 have a carbohydrate metabolism FUNCAT classification, but are also classified under valine, leucine, and isoleucine degradation under KEGG. The latter classification was accepted.

*These starred entries were significant in both experiment-1-QA response and experiment-2-starvation.

$ Leakiness in QA-expression is defined to be an absolute abundance of mRNA of >1000 on sucrose at time 0 and absolute abundance of mRNA on QA of >10,000 for a later time point than t = 0.

# significant at the 0.05 level.

### Searching for QA-1F binding sites in silico

Putative QA-1F-binding sites or *Quinic Acid-Response Elements* (QAREs) are identified with the program pattern (Accelrys, Inc.) operating on the 1000 nt upstream of each identified gene [47] from the file neurospora_crassa_#10BD2C.fasta derived from the Broad Institute Web site. The offset used is 1 and overhang 0. A mismatch of 2 is permitted. Patterns [19] searched for are:

GGATAATTATCC, GGRTAATTATCC, GGGTAA{4}TTATCC, GGATAA{4}TTATCC, GGGTAA{4}TTAAGC, GGTTAT{4}TCATCC, GGATGA{4}TTAACC, GGCTAA{4}TTAACA, GGGTAA{4}TTTTCC, GGCAAA{4}TCATCC, GGATAA{4}TAACCC, GGGGAA{4}TTATAG, GGATGA{4}TTCTCC, GGCGAA{4}TTACCC, CGTTAA{4}TTATTC, and GGCTCA{4}TCATCA.

### Ensemble methods

The ensemble method is used to fit genetic networks to the profiling data of experiment 1-QA response and prior published data (from a replicate of experiment 1-QA response) from Northerns on six of the seven *qa* gene cluster genes [2]. The protocol follows that detailed in the supplement to [11] with the following modifications. For each model tried, 20 random initial conditions are selected and for each initial condition a Markov Chain Monte Carlo is initiated with 35,000 equilibration sweeps and 5,000 accumulation sweeps. For each model, the initial condition, which results in the best (lowest χ^2^) in the MCMC run is used to initialize a second MCMC run of 40,000 accumulation sweeps. The solution method utilized for solving the trajectories for the ordinary differential equations (ODEs) is modified Euler since this used less time and gave comparable results to other methods [26]. The simulations were trivially parallelized by distributing the 20 independently initialized Markov chains over 20 independent processors. If the experimental data set comprises data from multiple experiments performed independently under different experimental control conditions, *e.g.*, due to different perturbations, a corresponding independent ODE system must be solved for each such experimental condition in every Monte Carlo updating step. Additional parallelization speed-up can then be achieved by distributing these independent ODE solutions over multiple processors. This ODE solver parallelization could be used in future simulations, as data from further experiments with different control conditions are added to the data set. ODE solver parallelization in the ensemble method was utlilized (but not capitalized on) in the simulations presented here, since the experiment 1-QA response data set comprises data from only a single experimental control condition.

## Results

### Is the control experiment needed in identifying QA-responsive genes?

The first question is whether or not the control experiment (*i.e*., a shift from sucrose to sucrose) has a significant effect on identifying QA-responsive genes. Previously we used a simple unpaired t-test to compare mRNA levels of cells grown on glucose to those grown on QA (Figure 2, [5]). A total of 895 genes with *QA upstream response elements* (QAREs) were identified as candidates for QA-responsive genes. As in Figure 5 of (1), the functions of responding genes were distributed over a broad array of functional categories including carbohydrate metabolism, ribsosome biogenesis, cell cycle, DNA metabolism, and others [5].

If instead we incorporated the control experiment and use here a paired t-test of differences in mRNA level (one of two places a traditional t-test is used in this paper for comparison with earlier work) between the control experiment and the experiment 1-QA response experiment (*i.e*., WT QA-response), then the number of responding genes in **Figure 2** is much more circumscribed in number. There are now only 300 genes in **Figure 2** responding to the shift in experiment 1-QA response when compared to the control experiment. Many of the same functional categories are seen, such as amino acid metabolism and carbohydrate metabolism in **Figure 2** as in Figure 2 of [5], but the number of identified responders is dramatically less. It is clear that the use of the control experiment has a significant effect on filtering for QA-responsive genes. We now turn to examining the effects of imposing the remaining predicted properties of QA-responsive genes with a multiple-test correction on the 895 initial candidates identified previously [5].

### What are the QA-responsive genes as identified by all four microarray experiments?

**Figure 3:**
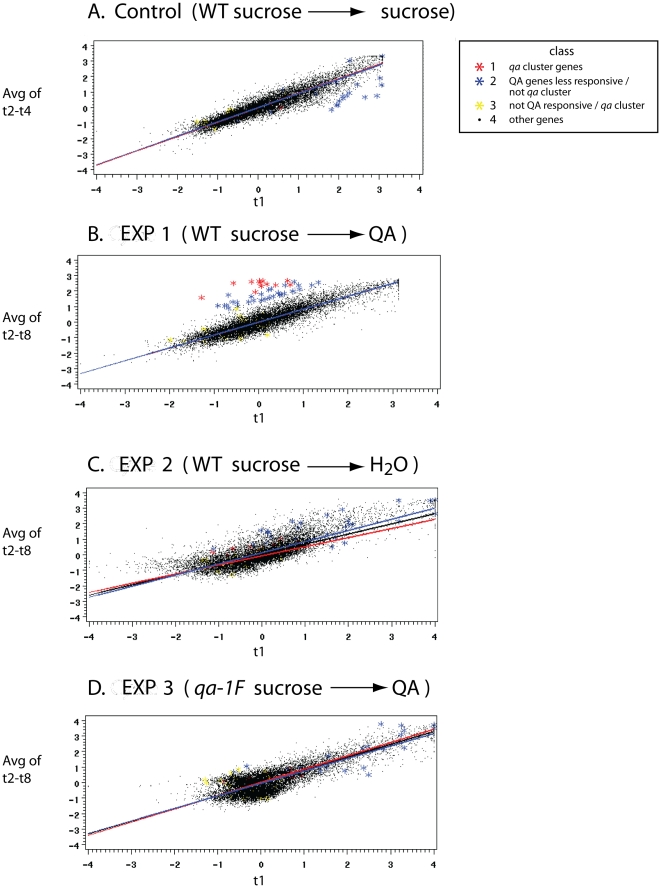
The average of background subtracted and normalized mRNA abundances of each gene at later times is regressed on its background subtracted and normalized mRNA abundance at time 0. Those genes that fall significantly above an average (in black) of the two regression lines (in red and blue) are used to characterize QA-responsive (panel B), starvation-responsive (panel C), and QA-responsive in a *qa-1F* mutant (panel D). The first panel A is a regression of an average of later time points of a particular gene on time point 0 for the same gene in the control experiment. Genes in red are *qa* cluster genes. Genes in blue are QA-responsive genes as defined in the text, which are not *qa* cluster genes. Genes in yellow are *qa* cluster genes not found to be QA-responsive (*i.e*., false negatives). Genes in black are all other genes. The regressions in other panels and color coding of genes are defined similarly for other panels B–D.

We now combine the results of all four microarray experiments in **Figure 3** to identify QA-responsive genes. First, the measured mRNA abundances over time are determined in each of four microarray experiments. The mRNA abundances are background subtracted and normalized as described in the Materials and Methods. Then the mRNA abundance at time 0 and the average abundance at a later time is normalized with a robust measure of scale to form a nontraditional t-statistic (see Materials and Methods). To determine if a significant change in mRNA abundance for each of the 11,000 genes in the *N. crassa* genome has occurred, a robust and linear regression of the t-statistic at later times (t_ave_) is performed on the t-statistic at time 0 (t_0_). It can be seen in **Figure 3** that the regression is quite good. If there is a significant increase in mRNA abundance by a particular gene in one of the four experiments, then its pair of values (t_0_, t_ave_) will fall far above an average of the two regression lines, after a suitable multiple-test correction using a significance level of 0.1 (see Materials and Methods). The significance test makes a normality assumption about the residuals (z) from the regression line in Materials and Methods and is examined in **Figure S2** for each experiment using histograms and normal plots [48].

Genes (in red) in **Figure 3** are known *qa* cluster genes that were identified as significantly responding in experiment 1-QA response; genes in yellow are known *qa* cluster genes that were not identified as significantly responding in experiment 1 (see Discussion). The remaining genes in blue are genes outside the *qa* cluster found to respond significantly in experiment 1-QA response, but not in experiments 2-starvation and 3-QA response *by qa-1F* or in the control experiment. A total of 50 features on the microarray chips are found to constitute QA-responsive genes after a multiple-test correction using a significance level of 0.10 [42]); see Materials and Methods and Table 1). Some of these QA-responsive genes (50) show up only as significant departures from the least median squares (LMS) regression line; a fewer number of QA-responsive genes (46) are identified by their departure from an average of the ordinary least squares (OLS) regression line and the LMS robust regression line (Table 2). Since some genes are represented in duplicate on the microarray chips, a total of 41 distinct genes can be found in Table 3 as QA-responsive. Most of these 50 genes are found to respond in experiment 1-QA response, but most not in experiments 2-starvation and 3-QA response by *qa-1F* and the control experiment. These genes also have the right mRNA kinetics (as is discussed below). These genes behave consistently with Prediction (1).

There is also an interesting set of QA-responsive genes (in blue) that fall significantly below the regression line in the control experiment (**Figure 3** – Panel A). Most of these genes appear to be transport genes, and they appear not simply to be off, but positively reduced in mRNA abundance in the absence of QA. It would appear that the cell does not waste resources producing these transporters when the metabolite is not available. This kind of observation on the transcriptional repression of QA related transporters has been seen for the *qa-y* gene (Figure 1 in [28]).

6 genes in the *qa* cluster were selected to validate the microarray analysis by Northern analysis using a replicate of microarray experiment 1 – QA response [2]. A total of 4 of these selected genes appear in Table 3, and 2 of these selected genes (*qa-1F* and *qa-x*) were selected as controls, having not appeared in Table 3. All six of these *qa* genes have 5–6 replicate microarray measurements per chip (see Materials and Methods). An analysis of covariance was performed using the log_2_ of the microarray expression level of each gene as the dependent variable and the log_2_ of the Northern expression level, as the independent covariate [49]. All slopes were significant at the 0.05 level except *qa-1F* (low abundance), and the slopes were all positive. For no gene could the parallel slopes hypothesis across replicate microarray measurements be rejected at the 0.05 level. The R^2^ for the 4 *qa* genes from Table 3 varied from 0.78–0.94; the R^2^ for the *qa-1F* and *qa-x* not from Table 3 were 0.13 and 0.48, respectively. The most highly expressed genes (*qa-2* and *qa-3*) had a very high R^2^ (>0.69). We conclude that 4 of the entries in Table 3 were validated by Northern analysis, but the *qa-1F* gene with low expression is an example of a gene that was missed in the microarray analysis (probably for reasons of power; see below).

The power and false positive rate of this test in experiment 1-QA response can be assessed empirically by the positive controls provided by *qa* cluster genes on each chip and also by 633 negative controls involving sequences from unrelated genomes (see Materials and Methods). An independently performed shift experiment to starvation conditions can be used to estimate the power in experiment 2-starvation [20]. The operating characteristics (*i.e*., false positive and false negative rates) of these experiments are summarized in Table 4. The power is lower than in previous microarray experiments on the clock (Table 1 in [16]).

**Table 4 pone-0020671-t004:** Observed fraction of false positives and false negatives among 633 negative controls on each microarray chip and among 40 distinct genes as positive controls using reported *qa* cluster genes.

Fractions observed					
Microarray Experiment	Nominal significance level (α)	False positives	False negatives	power	GEL_50_
QA-response by WT (Exp 1) (S/QA)	0.05	0.16	0.70	0.30	2.69
Starvation-response by WT (Exp 2) (S/Fries)	0.05	0.32	0.60	0.40	5.82
QA-response by *qa-1F* mutant (Exp 3) (turn QA-1F off on S/QA)	0.05	0.31	-	-	2.79

The estimated power is 1 – fraction of false negatives observed. The fraction of false positives observed can be compared with the nominal significance level used to identify genes that are: (1) QA-responsive in WT; (2) starvation-responsive in WT; (3) QA-responsive in *qa-1F*. The gene expression level-50 (GEL_50_) is a proxy for power [50] and allows comparison of power properties of the experiments below with other published experiments.

In addition, we calculated a proxy for power in Table 4 called the gene expression level −50 (GEL_50_) [50]. This GEL_50_ represents the fold variation between treatment (time points after the shift) and control (time point 0), at which 50% of the genes deemed to be significant have a larger fold variation than the GEL_50_. The values reported for experiment 1-QA response and experiment 3-QA response by *qa-1F* are in the range of at least 7 other published microarray experiments [50], [16]. The logistic regression used to calculate the GEL_50_ for experiment 3-QA response by *qa-1F* is only based on two positives (see Table 1), and the P-value of the slope in the logistic regression was P = 0.05.

### What genes have a starvation response?

**Figure 4:**
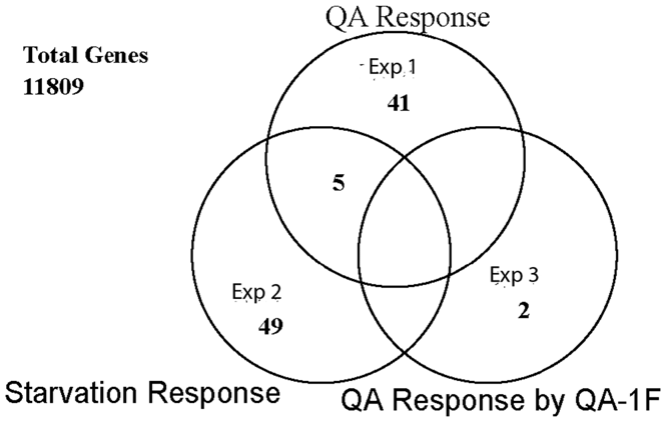
A Venn diagram summarizing the classification of all genes and control features on oligonucleotide arrays as QA-responsive (experiment 1), starvation-responsive (experiment 2), or QA-responsive in a *qa-1F* mutant (experiment 3). Any genes found to respond in the control experiment were subtracted from the counts in the Venn diagram. In the end a total of 11,809 features on the oligonucleotide arrays were classified as not responding in cycles 1, 2, or 3.

A total of 49 genes show a significant positive response to starvation in **Figure 4**. As shown in **Figure 4**, only 5 genes overlap in their response to starvation (i.e., shift from sucrose to Fries in experiment 2) with the QA-response (*i.e*., a shift from sucrose to QA in experiment 1). The small number of genes so identified would indicate that the cell appears to differentiate well between these two signals, quinic acid from no carbon source. In that so few genes [5] are elicited both in a starvation and QA-response, we have listed them in Table 3 (marked as starvation). So, 45 out of the 50 genes in Table 3 are consistent with Prediction (2).

Xie *et al*. [20] conducted a similar starvation experiment. In their experiment the shift was from 2% glucose to starvation conditions over a 2-hour window, and they only assayed 1335 genes on cDNA arrays with a two-color system, which is not as sensitive as the one-color system used here (see Materials and Methods). While they analyzed 8 data points per gene, we double this and analyze 16 data points per gene (8 under no shift from sucrose and 8 under shift to starvation conditions in Fries medium without a carbon source). To make the analysis of our microarray data comparable to that in [20], we perform a traditional paired t-test of mRNA abundance between experiment 2-starvation response and the control experiment to identify 1928 candidate genes involved in a starvation response (see paired t-test description in **Figure 2**). While Xie *et al.* [20] found 19% of the genes in the genome were involved in a starvation response based on a representative cDNA library, we find 18% of the genes implicated in a starvation response using microarrays representing all genes. We would expect to find that Xie *et al.* [20] would replicate 30% (from experiment 1-QA response) of the detected genes responding here to starvation from Table 2, and we observe 40% of the positives (Tables 1–5 in [20]) in agreement with the expectation from the experiment 2-starvation estimated power (30%) in Table 4. The experiment of Xie *et al.* [20] provides an independent validation of our microarray experiments here as well as an independent estimate of power for experiment 2-starvation in Table 4.

**Table 5:** Seven distinct ensembles are fitted to the profiles of all QA-responsive genes in Table 1 using a parallel implementation of the ens.f.

Model name	*qa* cluster genes (7) are controlled by:	QA-responsive genes (∼48) not in *qa* cluster are controlled by:	NCU3643 is controlled by:	# of theta-parameters	χ^2^ after 20×(35k Equilibration sweeps+5k accumulation sweeps)+40,000 sweeps in Accumulation run
1F-3643-2E A	QA-1F	QA-1F or NCU3643	QA-1F	147+239 = 386	1755.2
1F-3643-2E B	QA-1F	QA-1F or NCU3643	NCU-3643	147+239 = 386	1634.2
1F-3643-2E C	QA-1F	QA-1F or NCU3643	QA-1F or NCU3643	147+239 = 386	1666.0
2genes-2E	QA-1F	QA-1F(except ncu5897, *gla-1*)*	QA-1F	147+204 = 351	1707.4
all1F-2E(null hypothesis)	QA-1F	QA-1F	QA-1F	147+204 = 351	2026.3
all3643self-2E	QA-1F	NCU3643	NCU3643	147+204 = 351	1636.3
all3643-2E	QA-1F	NCU3643	QA-1F	147+204 = 351	1689.1

The regulatory networks among the 7 model ensembles differ by whether or not QA-1F or NCU3643 controls QA-responsive genes not in the *qa* cluster including NCU3643. The minimum goodness of fit χ^2^ over each ensemble is reported as well.

* This model, 2genes-2E, differs from model, all1F-2E, in shifting regulation of genes, ncu5897 and *gla-1*, to the control of NCU3643.

### Are there other pathways or regulators for metabolizing QA?

As a final prediction about QA-responsive genes, we would expect no response to shift to QA in a *qa-1F* mutant in experiment 3. If such a response were observed, it would be indicative of an alternate pathway for QA metabolism or an alternate regulator for *qa-1F*, as examples. Among the genes identified as responding to shift from sucrose to QA, two are found responding to the same shift in a *qa-1F* mutant. Given that 11,000 genes are tested, it is very likely that these 2 responders represent noise in the mRNA abundance data. The total number of genes responding to a shift from sucrose to QA in a *qa-1F* mutant in **Figure 4** is consistent with chance explaining the appearance of a significant response upon repeated testing, 11,000 times on 11,000 genes. We conclude that it is unlikely there is an alternate functional pathway for metabolizing QA or an alternate regulator of the *qa* cluster genes. Prediction (3) appears to be satisfied.

### What are the kinetics of mRNA abundance among QA-responsive genes?

**Figure 5:**
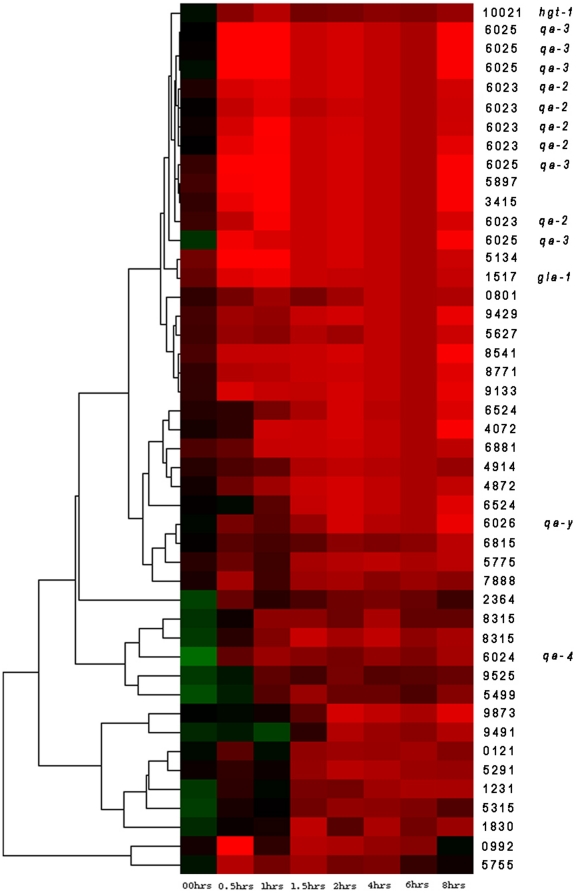
Transcriptional profiles of approximately 50 QA-responsive genes at 0, 0.5, 1, 1.5, 2, 4, 6, and 8 hrs after shift from sucrose (1.5%) to QA (0.3%) in experiment 1-QA response after background subtraction and normalization within arrays as described in Figure 4 and using Z_ijk_ (see Materials and Methods) in clustering with average linkage using the phenotypic correlation between mRNA profiles of different genes [44]. In this standard heat plot, black represents the starting value typically on sucrose, while degree of red is indicative of increased mRNA abundance on a log scale. Degree of green is indicative of a drop in mRNA abundance. **The bright green is −3, and the bright red is +3 is expression level on a base 2 log_2_ scale. Data arose from 8 chips probed with a biotin labeled aRNA. NCU numbers** [**47**] **and gene names (when available) are overlayed in the right margin of this heat plot. Genes in** **Fig. 1** **are represented at least 5 times on each chip. The 50 genes were selected by regressing an average mRNA abundance at later times on an mRNA abundance at time 0 for each gene in each of the microarray experiments. 50 genes fell significantly above the regression line (in black) in** **Figure 4** **experiment 1-QA response and not so experiments 2-starvation and 3-QA response by** ***qa-1F*** **usually and not in the control experiment and are listed in** **Table 3** **.**

The kinetics of the 50 QA-responsive genes are summarized in **Figure 5**. In **Figure 5** the positive response of all QA-responsive genes can be seen over time. These QA-responsive genes fall into two categories. In the top part of the heat plot, where most of the *qa* cluster genes are found (some in duplicate), the genes respond immediately to a shift to QA. On the bottom part of the heat plot in **Figure 5**, genes show a delayed responsive to QA. The genes with no delay fall principally into two functional categories; they are members of the *qa* gene cluster or they are sugar transporters usually.

**Figure 6:**
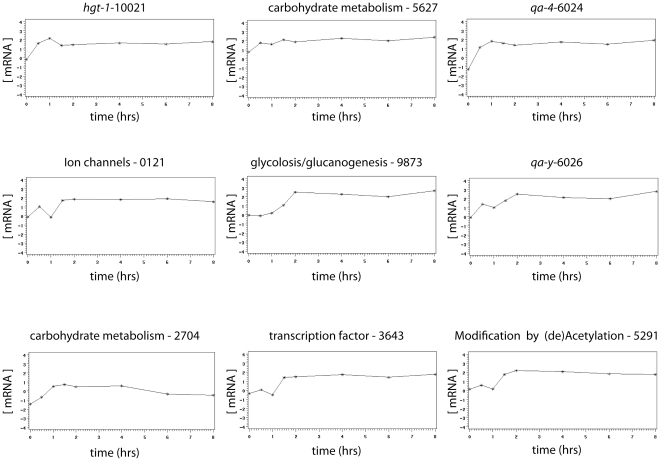
An exhaustive portrait of the shapes of responses to QA by QA-responsive genes. The 50 genes in Table 3 were visually sorted by JA into 9 categories as shown with one gene representing each category. Each RNA profile is a plot of mRNA abundance (background subtracted and normalized) for a particular gene over time in hrs. Each gene is labeled with its FUNCAT classification [43], its NCU number, and its gene name when available.

The detailed kinetics of QA-responsive genes are presented in **Figure 6**. This panel of temporal profiles of mRNA abundances for different genes is exhaustive of the kinds of temporal patterns observed among the 50 QA-responsive genes. We can see the fast response in mRNA abundance for the sugar transporter *hgt-1* and the *qa* cluster gene, *qa-4*, in **Figure 6**. There is also the more gradual response of the *qa-y* and a putative carbohydrate metabolism gene (NCU05627). The pronounced delay in kinetic response to QA can be seen in the transcription factor (NCU03643) and a gene (NCU09873) encoding a phosphoenolpyruvate carboxykinase involved in glycolysis/glucanogenesis [51]. The expectation for the kinetic response is concave down with time with a fall off in mRNA abundance over long enough time periods [2]. Some of the genes, such as NCU02704, show this kind of response. All of these genes are representative of the responses by the 50 genes in Table 3 and behave as expected from prediction (4).

### What are the functions of the QA-responsive genes?

**Figure 7:**
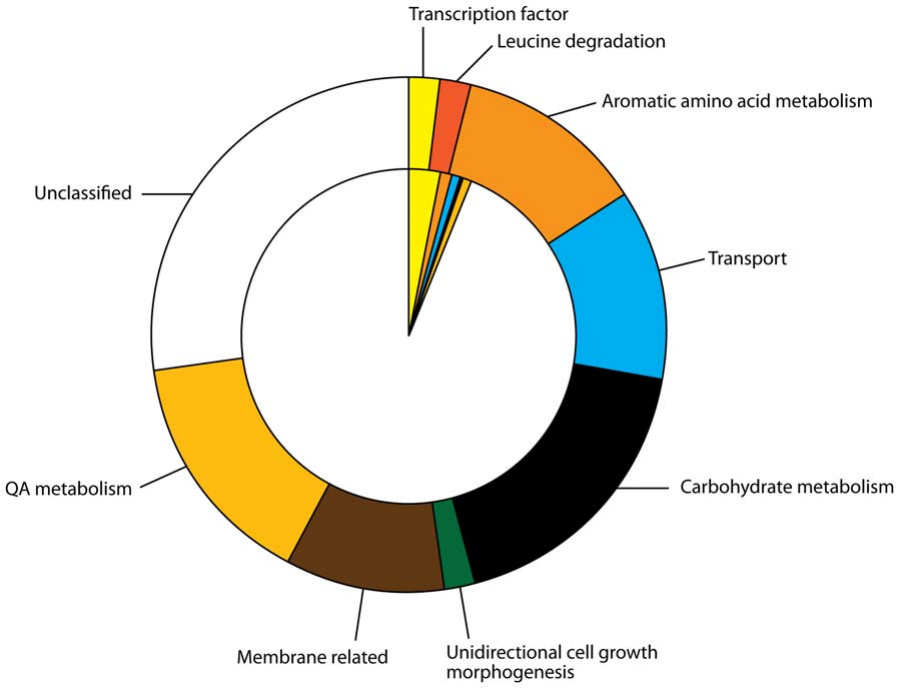
50 QA-responsive genes (as defined in the text) are classified by function (MIPS, [**43**]) in the outer wheel. Gene products of the *N. crassa* proteome are classified by function (MIPS, [43]) as well in the inner wheel. The definition of FUNCAT categories [43] is from Table 3: transcription factor other than QA-1F and QA-1S (11.02.03.04); leucine, valine, isoleucine degradation (1.05.01, 01.01.11.04.02); aromatic amino acid metabolism (1.01.09.05.02, NCU04072, NCU09429, 1.01.09.04.01); transport (20.01.03, NCU05775, 20.01.03.01); carbohydrate metabolism (1.05.01.01.01, 1.05.01.01.02, 2.01, NCU09429); unidirectional cell growth morphogenesis (40.01.03); membrane-related (16.09, 14.07.04, 20.03.01.01; QA metabolism (1.20, *qa* gene cluster encoded proteins; unclassified (99 and no KEGG assignment of function). The frequencies of the first 8 categories in the 50 QA-responsive genes vs. the frequencies in the genome were compared by an exact test facilitated with the use of Cochran's rules about the size of cell expectations [69]. For the exact test of homogeneity for this 2×8 table the P-value is 0.000059.

As a first step all 50 QA-responsive genes are assigned a MIPS (FUNCAT) classification in **Figure 7** [43]. In stark contrast to the broad array of functions linked with galactose metabolism in Figure 5 of [1], the QA-responsive genes are only distributed over 8 functional categories. This sharply defined distribution of function among QA-responsive genes is highlighted by a comparison with the proportion of these functions in the genome (inner wheel of **Figure 7**). It is also instructive to compare the collection of functions under QA-1F control with those under clock control because both exert regulation over carbohydrate metabolism. This focused collection of functions among QA-responsive genes is very different from the broad array of functions represented among *clock-controlled genes* in the biological clock of *N. crassa* [16] as well; moreover, in contrast to *clock-controlled genes*, QA-responsive genes are mostly of *known* function; only 27% of the QA-responsive genes are unclassified in function in **Figure 7**. Apparently the series of 4 microarray experiments are quite precise in selecting functions related to the *qa* gene cluster. The fact that the QA-responsive genes represent such a limited array of functions implies that the 27% of unclassified genes are likely to fall into this limited number of identified functional categories in Table 3.

**Figure 8:**
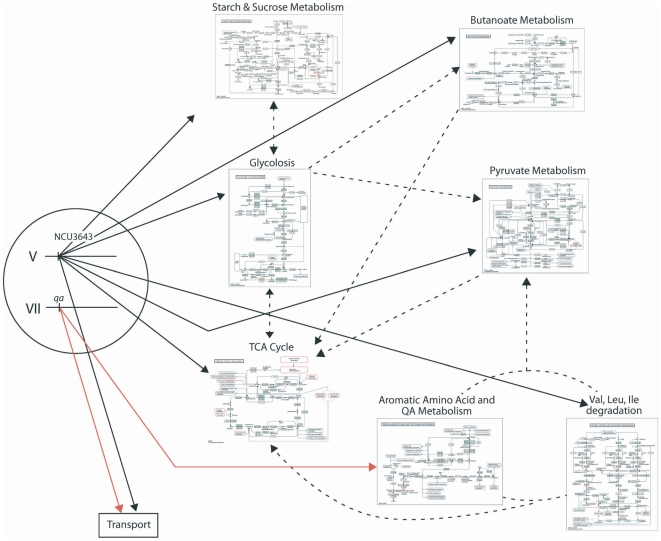
A model and portrait of the QA-responsive genes overlayed on the Kyoto Encyclopedia of Genes and Genomes (KEGG) pathways [**53**]. This model is consistent with either model all3643self-2E or all3643-2E in Table 5, depending on what is assumed about how NCU3643 is regulated. Arrows in red indicate transcriptional control by QA-1F. Arrows in black indicate transcriptional control by the “activator” NCU03643 on linkage group V [31]. The metabolic modules from KEGG are thumbnail sketches and can be clicked on for their enlargement. Enzymes outlined in red or in red in pathways are encoded by QA-responsive genes. Metabolic modules in red are thought be under QA-control. Connections by dotted arrows between metabolic modules are hypothesized by KEGG. The pathways themselves are derived from biochemical studies on a variety of species. Thumbnails can be enlarged for Starch and Sucrose Metabolism (Figure S4), Butanoate Metabolism (Figure S5), Glycolysis (Figure S6), Pyruvate Metabolism (Figure S7), TCA Cycle (Figure S8), Aromatic Amino Acid and QA Metabolism (Figure S9), and Val, Leu, Ile degradation (Figure S10).

When the QA-responsive genes are overlayed on the Kyoto Encyclopedia of Genes and Genomes (KEGG) pathways [52]–[53], the 8 functional categories represented in the QA-responsive genes are tightly distributed across 8 interconnected metabolic modules, all linked with the TCA cycle or glycolysis, as shown in **Figure 8**. The QA metabolism pathway has long been known to be functionally coupled to aromatic amino acid metabolism [25] and has been hypothesized to feed into the TCA Cycle [54]. As predicted [25], aromatic amino acid metabolism is well-represented (12%) among the 50 QA-responsive genes in **Figure 5** (top cluster). All of the metabolic modules with QA-responsivxe genes are directly connected to the TCA cycle, except for the starch and metabolism module, and a number of enzymes in aromatic amino acid metabolism are encoded by QA-responsive genes.

QA-responsive genes in aromatic amino acid biosynthesis tend to be part of the *qa* cluster (but see NCU03415, NC01830, NCU05499, and NCU04072), and aromatic amino acid biosynthesis is coupled to Val, Leu, and Isoleucine degradation through 4 QA-responsive genes (NCU03415, NCU06881, and NCU02704, and NCU00591). The gene NCU02704 encodes a putative 2-oxoisovalerate dehydrogenase E2 component, which may control flow through all three pathways in the Val, Leu, & Ile degradation module in **Figure 8**. The gene NCU06881 encodes a putative succinyl-CoA:3-ketoacid-coenzyme A transferase subunit A possibly linking the Val, Leu & Ile degradation module to butanoate metabolism in **Figure 8** as well as possibly controlling metabolic flux to a key metabolite, Acetyl-CoA. Prediction (5) is satisfied.

From prior work (M. E. CASE, unpublished) it was predicted (*i.e*., Prediction (6)) that sorbose resistant mutants involved in starch and sucrose metabolism would involve QA-responsive genes because sorbose-resistant mutants constitutively activate QA-metabolism on sucrose as discussed earlier. The only metabolic module with a QA-response gene two steps removed from the TCA cycle is the *gla-1* (NCU01517) gene inferred to encode a glucoamylase precursor converting glycogen or dextrin to α-D-glucose. The gene *gla-1* has the same biochemical activity as the *gla-2* (also known as *sor-4*) gene, which as a mutant has a sorbose-resistant phenotype. This gene can be thought of as encoding the last step in glucanogenesis or the first step to metabolizing an energy reserve.

The TCA cycle contains a QA-responsive gene (NCU09873); this gene encodes a phosphoenolpyruvate carboxykinase, which produces phosphenolpyruvate (PEP), another key metabolite leading to (into) the TCA cycle, glycolysis/glucanogenesis, and pyruvate metabolism [14]. By conversion of PEP to pyruvate there is also a link to Val, Leu, and Ile degradation. This phosphoenolpyruvate carboxykinase encoding gene also has an upstream QARE for regulation by QA-1F (see Table 3). It has also been identified as being elicited by a starvation response in *N. crassa* [20] and here in experiment 2-starvation.

The gene (NC03415) encoding a putative aldehyde dehydrogenase is QA-responsive and links glycolysis and glucanogenesis to a number of other metabolic modules in **Figure 8**, including the TCA cycle, Val, Leu, and Ile degradation, aromatic amino acid metabolism, pyruvate metabolism, and butanoate metabolism. This enzyme appears to be a major coupler of metabolic modules in **Figure 8**. The organisms may carry out this flux control of multiple metabolic modules by metabolizing acetate to and from acetaldehyde. For example, this enzyme appears to control flux in all three metabolic modules by converting products of alcohol dehydrogenase into Acetyl-coenzyme A (coA) (connecting to the TCA cycle, pyruvate metabolism, and glycolysis/glucanogenesis) in **Figure 8** [14]. This gene also has a QARE upstream (see Table 3).

The connection to butanoate metabolism appears to be largely redundant with respect to enzymes found in other metabolic modules, namely the NCU06881 encoding a putative succinyl-CoA:3-ketoacid-coenzyme A transferase subunit and the NCU03415 encoding a putative aldehyde dehydrogenase previously discussed. Both of these enzymes control flow to acetoacetate for entry into and out of butanoate metabolism.

The links to pyruvate metabolism include the previously discussed NCU09873 gene encoding a phosphoenolpyruvate carboxykinase controlling flow into the pyruvate metabolism module and the putative aldehyde dehydrogenase encoded by NCU03415 controlling flow through the module.

The last metabolic module includes the sugar and amino acid transporters. The *hgt-1* gene is reported as responding significantly to a starvation response [20], and we also find it to respond to starvation and QA in **Figure 5**. Among the 90 transporters in the *N. crassa* genome [43], there are 9 such transporters that are QA-responsive in Table 3; one of these is thought to be an amino acid transporter (NCU05775). Another is thought to be a lactose transporter (NCU00801).

### Ensemble fitting of 7 distinct genetic networks to the profiling data from the different microarray experiments

In this section we specifically test how the QA-responsive genes in Table 3 are regulated. We used the ensemble method of network identification [2], [55]–[58]. In Table 5 we fitted several extensions of the genetic network in **Figure 1** with 147 molecular species and 204 reactions for the null hypothesis (denoted all1F-2E). The extensions vary as to what (QA-1F or NCU03643) regulates the QA-responsive genes outside the *qa* cluster. For example, one model entertained is that only QA-1F regulates the QA-responsive genes (all1F-2E). In another model only NCU03643 regulates QA-responsive genes outside the *qa* cluster and itself (all3643self-2E) as shown in **Figure 8**. In order to give ourselves a more extensive exploration of the parameter space, each model ensemble was pursued for 40,000 sweeps (35,000 equilibration sweeps+5,000 accumulation sweeps) in parallel to identify the best candidate among 20 different random initial conditions for the MCMC Method (see Materials and Methods and **Figure S3**), which is then used to initiate a final accumulation run of 40,000 sweeps.

**Figure 9:**
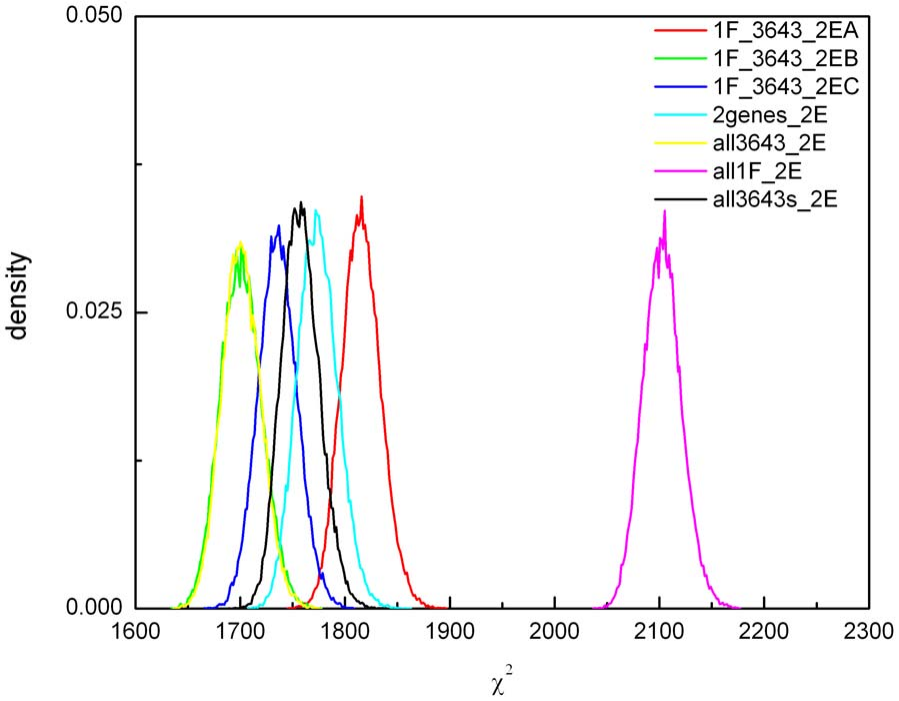
The χ^2^ distributions differ (do not overlap) for the six alternate hypotheses (Table 5) from (with) that of the null hypothesis (all1F-2E). Equilibration and accumulation runs of the ensemble method used to calculate these distributions are described in Materials and Methods. The label all3643s-2E is an abbreviation for model all3643self-2E in Table 5.

The results would indicate that the simplest (null) hypothesis that only QA-1F regulates the QA-responsive genes can be rejected in favor of any of the six alternative hypotheses in Table 5. The alternative hypothesis in **Figure 8** (all3643-2E) has a lower minimum χ^2^ than the null hypothesis (1489 vs. 1754 in Table 5). The only ensemble, which can be rejected, is the null hypothesis (all1F-2E) in which QA-1F regulates all QA-responsive genes and the *qa* cluster). The remaining models are not distinguishable based on the profiling data so far accumulated. The distribution of chi-squared values across 7 model ensembles is shown in **Figure 9**. The overlap in these distributions is another indication that the alternate models tried cannot be distinguished. The first three models in Table 5 contain the null hypothesis, and so an ordinary difference of the χ^2^ s can be used with df = 35 and α = 0.05. These differences are significant at at least the 0.001 level. We conclude that the delay in expression of some QA-responsive genes is explained in part by the regulation by NCU03643.

**Figure 10:**
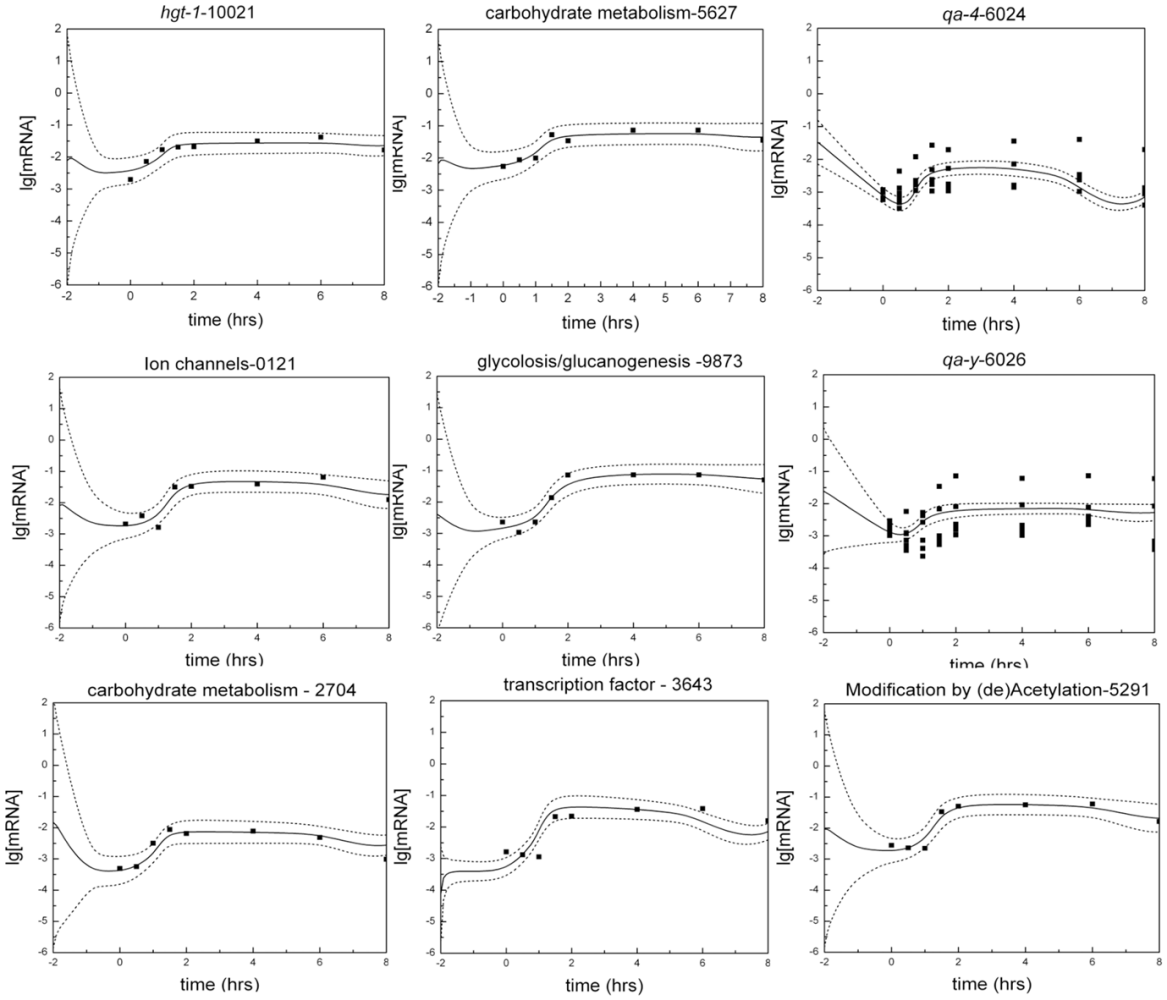
The ensemble of models, all3643-2E, well predicts the dynamics of all genes in the network. As examples, the genes in Fig. 8 with very different dynamics are plotted with log-concentration on the y-axis and time in hours on the x-axis. The solid lines are the ensemble means on a log-scale. The dashed lines are +/− two standard deviations across the ensemble. The dots are microarray or Northern data. The numbers at the top of each box are the first four digits of an NCU number.

In **Figure 10** we show how the ensemble for model all3643-2E predicts all the major classes of responses to shift to QA. The predictions are quite in accord with the profiling data. The only issue is that the profiles of *qa-4* and *qa-y* are a little over-dispersed as was the case for the clock network [16]. The *qa-4* and *qa-y* data in **Figure 10** include the Northern data from a replicate of experiment 1-QA response to validate the microarray data reported [2].

### Use of markers for a QA-response in other studies

It is common practice to include a *qa-2* promoter in various expression vectors to study other processes in *N. crassa* (see for example, [59]); [16]). In hooking up a gene to a *qa-2* promoter it would be useful to have an independent positive control for a response to quinic acid (QA). In principle, any of the 50 genes in Table 3 could be used, but it would be highly-desirable to use a gene that is truly off (not leaky), when there is no QA in the medium.

In Table 3 the maximum and minimum absolute abundance measured over an 8-hour window from sucrose to QA is recorded from experiment 1-QA response. *Leakiness* is defined to be an absolute mRNA abundance above 1000 on sucrose. In Table 3 four genes are not leaky (in bold), and have an absolute mRNA abundance on QA of at least 10,000 on a 0–64,000 scale. In Table 3 genes that would represent good positive controls with little leakiness would be *qa-4*, *fea-1*, *hgt-1*, and NCU01231. The QA-responses of *qa-4* and *hgt-1* are shown in **Figure 6**.

## Discussion

### How is the shift to quinic acid predicted to affect the flow through metabolic modules?

Work on other systems, such as *S. cerevisiae*, has identified enzymes that are key to determining the flow through metabolic modules in **Figure 8** [14]. As an example, the putative aldehyde dehydrogenase encoding gene (NCU03415) may determine flow in several metabolic modules including to the TCA and Glyoxylate Cycles. This would suggest that aldehyde dehydrogenase could have a particularly important role in flux through the TCA cycle upon shift to QA. We expect that by the up-regulation of the putative aldehyde dehydrogenase as a direct response to shift to QA would increase flux through the TCA and glyoxylate cycles. In contrast in prior work [20] starvation was found to have relatively little effect on the components of the TCA cycle.

Another key enzyme in control of flux in glycolysis/glucanogenesis was the QA-responsive gene (NCU09873) encoding a phosphoenolpyruvate carboxykinase [14]. This enzyme may control the direction of flow in glycolysis/glucanogenesis under glucose starvation. When induced under QA here, the expectation is that flow would be in the direction of glucanogenesis. Under quinic acid carbon source or starvation the organism is storing carbon and energy in glucanogenesis. This is similar to the flow under glucose starvation [14] and opposite that implied by [60] on galactose (GAL) in *S. cerevisiae*, where GAL feeds into glycolysis. The enzyme (NCU09873) may also control flow to pyruvate metabolism and Val, Leu, and Ile Degradation modules as well.

QA-responsive genes are distributed extensively in aromatic amino acid metabolism and Val, Leu, and Ile degradation. The two modules appear connected in a cycle in **Figure 8**. From the strong QA-response of the putative 2-oxoisovalerate dehydrogenease E2 component, we hypothesize that back flow through this degradation pathway is increased on shift to QA. All three pathways in this module are affected by this one enzyme, 2-oxoisovalerate dehydrogenease.

While these flux predictions can be made, the caveat is that measured fluxes in metabolic pathways don't always reflect what is predicted from measured expression levels (the basis of our flux predictions) [61]. What is needed is to measure the fluxes to test these predictions [62]. Then an integrated approach combining measured fluxes with expression data could be enacted as in the study of C(1) metabolism in *Methylobacterium extorquens* [63].

### Discovering the connections in a genetic network

One of the striking results of the work by [1] is the large number of genes (997) responding to a shift from raffinose to galactose over a broad array of functions as in **Figure 2** for the *qa* gene cluster. It came as somewhat of a surprise that such a well-studied system as the *GAL* genes could have such ramifying effects in the cell. The challenge to understanding this result is that a transcription factor, such as Gal4p or QA-1F, only has a limited number of targets [21]–[22]. Ren et al. [60] identified only 10 targets for GAL4p. The shift experiments in [1] or [5] did not include a number of controls that are used here to identify QA-responsive genes in **Figure 3**. A simple control, such as a shift from sucrose (1.5%) to sucrose (1.5%), in conjunction with the experiment 1-QA response microarray experiment of shift from sucrose (1.5%) to QA (0.3%) has a dramatic effect on the number of genes identified as QA-responsive, as shown in **Figure 2** when compared with earlier work (Figure 2 in [5]).

Using a genetic network model in **Figure 1** for how the *qa* gene cluster functions, a number of predictions can be made about how a QA-responsive gene should behave (see predictions in Introduction). This behavior can be used to filter for QA-responsive genes. When such a filter is applied in **Figure 3**, a strikingly different result is obtained from that in [1]. Only 50 QA-responsive genes are identified as might be expected from the average number (38) of binding sites per transcription factor in *S. cerevisiae* [21]–[22]. The array of functions among QA-responsive genes is also telescoped as shown in **Figure 8** to functions closely connected with QA metabolism. When the diauxic shift is examined under deletion of the regulation *TUP1*, for example, a similar targeted response to diauxic shift in *S. cerevisiae* is reported [14]. In conclusion, the controls introduced into the microarray experiment are critical to identifying genes directly affected by shift to QA from other ancillary effects, such as starvation.

### A genetic network for QA metabolism

In earlier work a simple model was developed for the *qa* gene cluster [2]. A series of model-guided microarray experiments have been carried out to place this genetic network in its larger metabolic context [5]. Six predictions from the model and earlier work were largely validated. A fairly detailed hypothesis is fleshed out in **Figure 8** including one new cutinase transcription factor-1β (NCU03643) of previously unknown function [31] as well as eight metabolic modules coupled to the core QA pathway in **Figure 1**.

The cutinase transcription factor (NCU03643) is in the fungal binuclear cluster Zn(II)2Cys6 family, the largest family of transcription factors in *N. crassa*, of which there are 77 members [64]. This family includes Gal4p and QA-1F, as examples. While a knockout of this transcription factor (NCU03643) had no obvious phenotype [31], the microarray experiments summarized here in **Figure 3** yield up several phenotypes. The transcription factor NC03643 is QA-inducible and is starvation responsive. A mutation in *qa-1F* suppresses the QA-induction of NCU03643, so NCU03643 is epistatic to *qa-1F*. Both Dong et al. [16] and Chen *et al.* [65] reported that NCU03643 is light-responsive. The series of experiments here help to establish the first functional annotation of this transcription factor. This transcription factor appears to provide an assist to QA-1F in adapting to a shift to QA as hypothesized in **Figure 8** based on the NCU03643 phenotypes in **Figure 3**, and based on its phenotypes we hypothesize NCU03643 is one of the few targets of QA-1F outside the *qa* cluster. In **Figure 5** (*i.e*., the heat plot) we also identify potential targets of NCU03643 with a delayed response to shift to QA. The delayed response of NCU03643 induction in **Figure 6** may help to explain those genes with a delayed QA-induction in **Figure 5**, as supported by ensemble fitting of a genetic network in which both QA-1F and the protein encoded by NCU03643 regulate QA-responsive genes (Table 5).

A second novel feature of the model in **Figure 8** is the involvement of Valine, Leucine, and Isoleucine degradation in the QA-response. The Val, Leu, & Ile degradation module appears coupled to the aromatic amino acid metabolism module, which has long been known to be affected by QA metabolism. For example the *aro-9* gene which encodes a product in the Shikimate pathway is functionally redundant with the product of *qa-2* [66]. These two amino acid metabolism modules are connected in a metabolic cycle. One of the findings of the microarray experiment is that a gene (NCU02704) encoding a putative 2-oxoisovalerate dehydrogenase E2 component found in all three pathways of this amino acid degradation module is strongly up-regulated in **Figure 8**. In fact, three other genes with products on this pathway are also up-regulated in **Figure** 8. These two amino acid metabolism modules are coupled to the TCA cycle through Acetyl-CoA and through Succinyl-CoA. A complete picture of QA metabolism thus appears to require particular elements of amino acid metabolism, namely the aromatic amino acid and Val, Leu, & Ile degradation modules.

A final interesting new feature of the revised model of QA metabolism in **Figure 8** is the inclusion for the first time of genes outside the *qa* gene cluster under the apparent direct control of QA-1F or possibly the gene (NCU03643) and those in the transport module. Also for the first time the up-regulation of a variety of transporters other than *qa-y*, such as the high-affinity glucose transporter *hgt-1* in **Figure 6**, appears to be involved in allowing QA into the cell. Many of these transporters appear to have the telltale signs of being high-affinity QA transporters because they are positively down-regulated in the control experiment (See **Figure 3**, Panel A), as has been reported for the *qa-y* gene [28]. This control of other transporters outside the *qa* cluster is also found in the *GAL* genes [60], albeit negative regulation of other transporters in the case of *PCL10* in *S. cerevisiae*.

### Limitations of the microarray analysis in Figure 3

The most severe limitation of the microarray analysis in **Figure 3** is the power to detect QA-responsive genes in Table 4. While the experiments here more than double in size previous experiments [20] and in line with the power properties of other microarray experiments [50], the power to detect QA-responsive genes is still low. It is likely that larger replicates of the experiments here will detect further QA-responsive genes. Some genes that are likely to be found are those encoding isocitrate lyase (NCU04230) and malate synthase (NCU10007) in the glyoxylate cycle [5].

One of the critical variables that affects the power of these microarray experiments is the adequacy of the oligonucleotide tags for each gene on the arrays. We made an effort to choose two distinct tags for each of the *qa* cluster genes with the exception of *qa-2*. The tag for *qa-2* had been used successfully previously [16]. What we found is that only one of the two distinct tags for *qa-4* and *qa-y* shows a clear response to QA. We had observed this effect of distinct tags on a gene response in prior work for the gene *wc-1* [16]. If we had duplicated the “good” tag on *qa-4* and *qa-y* as described in Materials and Methods, we would have had a much higher power estimate more comparable to that in Table 3 of [16]. It appears planning some redundancy in oligonucleotide tag selection on arrays for genes is recommended.

Another limitation of the experiments here is that while it would have been ideal to have the microarray experiments in all four experiments in **Figure 3** done on the same scanner (See Materials and Methods), this was not possible. So, we have relied on a test for QA-responsive genes in **Figure 3** that does not involve relying on comparisons of mRNA abundances between experiments to identify QA-responsive genes.

### Comparison of different system biology approaches to understanding carbon metabolism

The model in **Figure 1** is similar to the metabolic network developed for *E. coli* for carbon metabolism [3] with at least 436 species and 720 reactions currently.The genetic network for QA metabolism was started with 38 species and 54 reactions in **Figure 1** and been expanded to include the 50 QA-responsive genes and their products in **Figure 8** for a total of 147 species and 204 reactions. Both of the genetic networks in *E. coli* and *N. crassa* began with a core metabolic module, such as **Figure 1** or **8**, which included carbohydrate and amino acid metabolism [67], [2]. The end goal is the same, a complete description of the metabolism of a carbon source.

Both teams begin with an iterative model-guided discovery process as in Figure 2 of [16] or in Figure 1 of [3]. From here the approaches diverge. No shortcuts are being taken here. Here the regulatory component introduced by genes has not been approximated with a “logic statements to simulate regulatory processes” [3]. No quasi-steady approximation is being invoked to carry out a Flux Balance Analysis [67], [3]. Instead a full dynamic solution is being sought using methods drawn from statistical mechanics capitalizing on ensemble methods for genetic network identification to obtain bulk behavior of the network [2], [5]. In order to implement a full dynamic solution it was helpful to parallelize the ensemble method of network identification as described in the Materials and Methods. The differences in these two approaches is well described in Figure 1 of [68].

## Supporting Information

Figure S1 **RNA profiles of experiment 1-QA response samples by RNA Nano LabChip (Agilent Technologies, Inc.).** The leftmost lane is an RNA ladder with marker sizes indicated in nucleotides. Successive time points are indicated in hrs at the bottom of each lane.
(TIFF)

Figure S2 **Distribution of residuals from the regression line (in black) after background subtraction and normalization.** Gene features with positive residuals are considered above background (see Materials and Methods). Responders are those in the right tail of each distribution shown. In each panel the inset is a normal plot [48] in which observed ranks of residuals (y-axis) are plotted against their expected ranks (x-axis) from a normal distribution. Linearity is indicative of normality. Each of the panels A–D correspond to the panels (experiments) in Fig. 4.
(TIFF)

Figure S3 **3 of the 20 give random initial conditions give similar fits as measured by the χ^2^_χ_.** The remaining 17 random initial conditions do slightly worse. Each of the 20 equilibration runs was done for 35,000 sweeps followed by an accumulation run of 5000 sweeps for a total of 40,000 sweeps. All 20 runs involve a total of 200,000 sweeps. The ensemble with the best (lowest) χ^2^ (*i.e*., run 1) was used to initialize an accumulation run of ∼40,000 sweeps to construct the final ensemble for each network tried in Table 5. The network displayed is the null hypothesis, all1F-2E, from Table 5. In this example the first random initial condition tried led to the best final fit.
(TIF)

Figure S4 **KEGG pathway for starch & sucrose metabolism** [**53**] **.** Enzymes in red are encoded by QA-responsive genes in Table 3.
(TIF)

Figure S5 **KEGG pathway for butanoate metabolism** [**53**] **.** Enzymes in red are encoded by QA-responsive genes in Table 3.
(TIF)

Figure S6 **KEGG pathway for glycolysis** [**53**] **).** Enzymes in red are encoded by QA-responsive genes in Table 3.
(TIF)

Figure S7 **KEGG pathway for pyruvate metabolism** [**53**] **.** Enzymes in red are encoded by QA-responsive genes in Table 3.
(TIF)

Figure S8 **KEGG pathway for TCA cycle** [**53**] **.** Enzymes in red are encoded by QA-responsive genes in Table 3.
(TIF)

Figure S9 **KEGG pathway for aromatic amino acid and QA metabolism** [**53**] **.** Enzymes in red are encoded by QA-responsive genes in Table 3.
(TIF)

Figure S10 **KEGG pathway for Val, Leu, Ile degradation** [**53**] **.** Enzymes in red are encoded by QA-responsive genes in Table 3.
(TIF)
