# Supplementary figures and images for: Systems Biology of the qa Gene Cluster in Neurospora crassa
Source: PLoS One. 2011 Jun 14;6(6):e20671. doi: 10.1371/journal.pone.0020671 (PMC3114802; doi:10.1371/journal.pone.0020671)

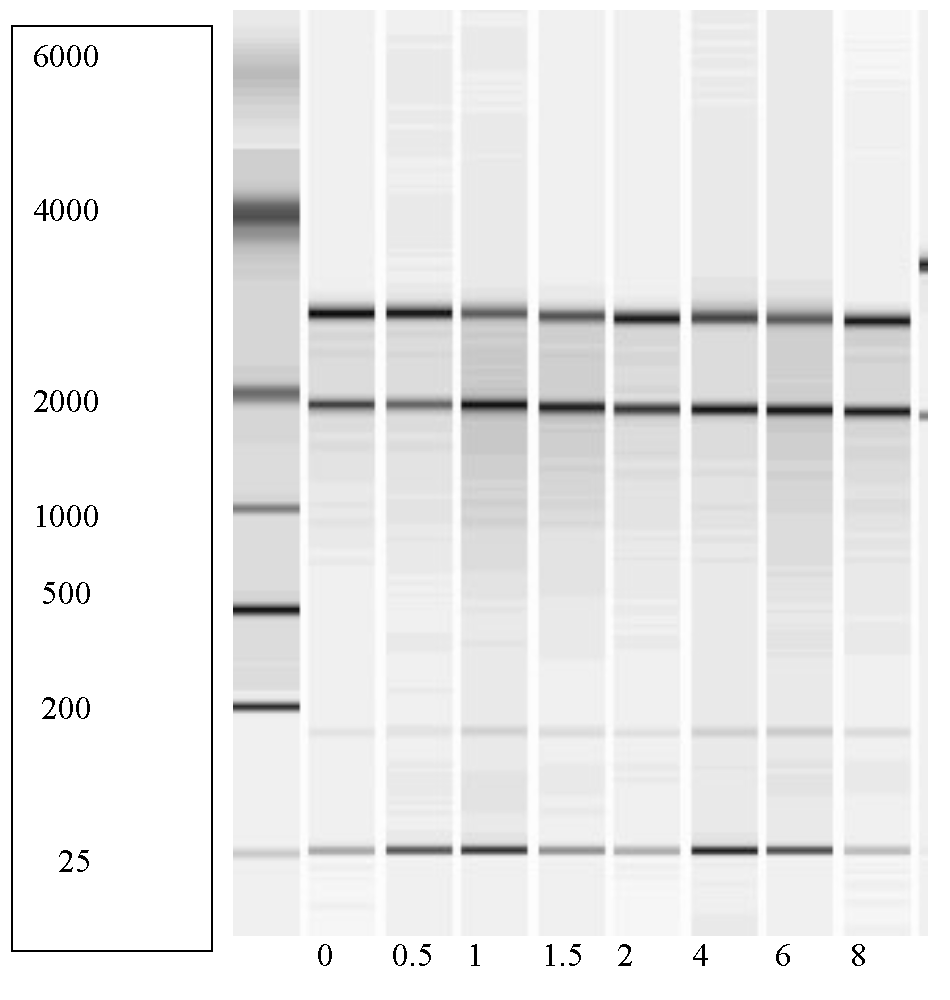

Supplement: Figure S1 — RNA profiles of experiment 1-QA response samples by RNA Nano LabChip (Agilent Technologies, Inc.). The leftmost lane is an RNA ladder with marker sizes indicated in nucleotides. Successive time points are indicated in hrs at the bottom of each lane. (TIFF) [file pone.0020671.s001.tiff]

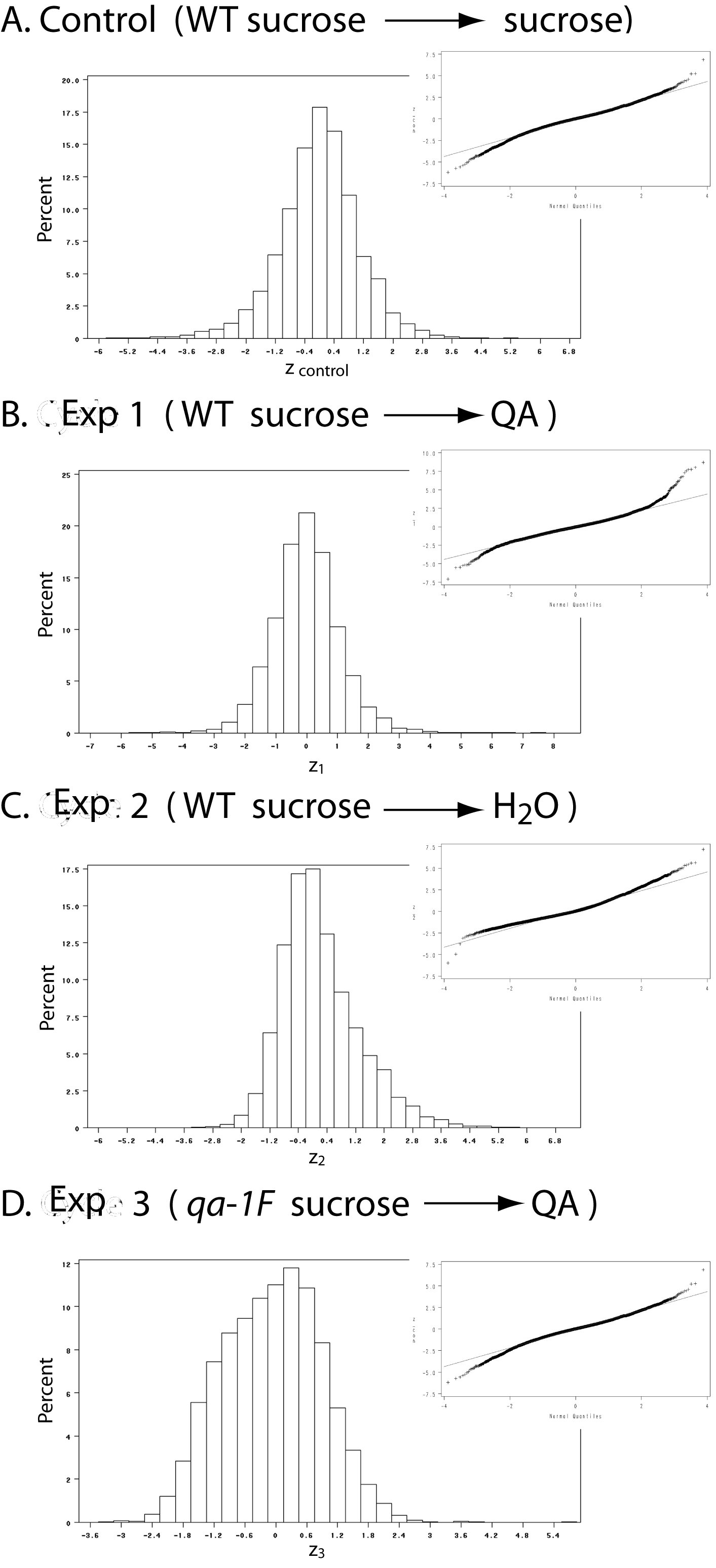

Supplement: Figure S2 — Distribution of residuals from the regression line (in black) after background subtraction and normalization. Gene features with positive residuals are considered above background (see Materials and Methods). Responders are those in the right tail of each distribution shown. In each panel the inset is a normal plot [48] in which observed ranks of residuals (y-axis) are plotted against their expected ranks (x-axis) from a normal distribution. Linearity is indicative of normality. Each of the panels A–D correspond to the panels (experiments) in Fig. 4. (TIFF) [file pone.0020671.s002.tiff]
